# Microsurgical technique for concurrent cervical corpectomy and ventral phrenic nerve access in the rat

**DOI:** 10.1016/j.mex.2018.08.007

**Published:** 2018-08-13

**Authors:** Michael G.Z. Ghali

**Affiliations:** Dept. of Neurobiology & Anatomy, Drexel University College of Medicine, 2900, Queen Lane, Philadelphia, PA, 19129, USA

**Keywords:** Corpectomy with concurrent ventral phrenic nerve access, corpectomy, ventral approach, spinal cord

## Abstract

The phrenic nerve is useful to record as a motor output in studies investigating neural control of respiration. It may be accessed via dorsal or ventral microsurgical approaches. Since such studies frequently involve concurrent access to the spinal cord, the two approaches may be alternatively used, each with its own set of advantages and disadvantages. The dorsal approach permits easier exposure of the spinal cord via laminectomy, but, compared to the ventral approach, phrenic nerve access proves more challenging, and concurrent surgical access to the full complement of respiratory-related nerves (i.e., glossopharyngeal, vagus, recurrent laryngeal, hypoglossal nerves) and cervical sympathetic nerve in the neck is limited. The ventral approach achieves more direct access to the phrenic and respiratory-related nerves, but ventral access to the spinal cord via corpectomy requires much greater diligence and vigilance. Ventral spinal cord access, however, facilitates neuronal (e.g., phrenic motoneuron and interneuron) recordings in the ventral horn of the spinal cord, given greater proximity to the ventral compared to the dorsal surface of the spinal cord providing more leeway in recording pipette insertion point and trajectory. Additionally, ventral access to the cervical spinal cord proves useful across a broad range of studies investigating normal spinal cord physiology as well as spinal cord injury. We detail the microsurgical technique of concurrent ventral phrenic nerve dissection and cervical corpectomy in adult rats.

## Graphical abstract

**Figure:**
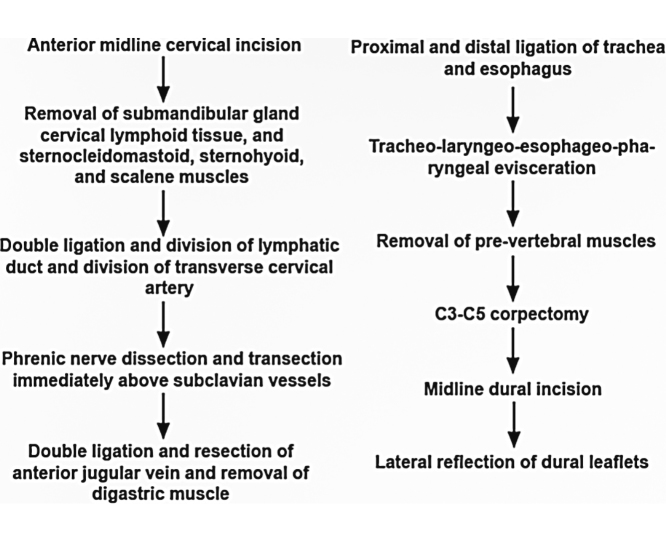

**Specification Table**

**Table:** 

Subject Area	Neuroscience
More specific subject area	Microsurgery
Method name	Corpectomy with concurrent ventral phrenic nerve access
Name and reference of original method	Castro-Moure F, Goshgarian HG. Reversible cervical hemispinalization of the rat spinal cord by a cooling device. Exp Neurol 141: 102–112, 1996.
	Castro-Moure F, Goshgarian HG. Morphological plasticity induced in the phrenic nucleus following cervical cold block of descending respiratory drive. Exp Neurol 147: 299–310, 1997.
	Marchenko V, Ghali MG, Rogers RF. Motoneuron firing patterns underlying fast oscillations in phrenic nerve discharge in the rat. J Neurophysiol 108: 2134–2143, 2012.
	Marchenko V, Ghali MG, Rogers RF. The Role of Spinal GABAergic Circuits in the Control of Phrenic Nerve Motor Output. Am J Physiol Regul Integr Comp Physiol. 308:R916-R926, 2015.
	Marchenko V, Rogers RF. GABAAergic and glycinergic inhibition in the phrenic nucleus organizes and couples fast oscillations in motor output. J Neurophysiol 101: 2134–2145, 2009.
Resource availability	Sigma Aldrich

## Method details

Ventral access to the cervical spinal cord proves useful in many experimental investigations. This approach allows access to the phrenic nerve and other respiratory-related nerves in the anterior cervical region, including the glossopharyngeal, superior and recurrent laryngeal, vagus, and hypoglossal nerves. It also allows access to carotid artery and internal jugular vein, allowing ligation of the internal carotid artery in preparation for decerebration. Contemporaneous access is provided to the ventral surface of the cervical cord, facilitating phrenic nerve and phrenic motoneuron recordings. This approach, however, entails unique challenges. We detail the microsurgical technique for cervical corpectomy and ventral spinal cord access, which has been described in previous reports [[1], [2], [3], [4], [5]].

## Results

Phrenic nerve access and corpectomy were successfully performed in all animals without complications. Animals had good hemodynamics (normotensive to slightly hypertensive) and regular respiratory rhythm and pattern. Recordings of phrenic nerve and phrenic motoneurons with good signal-to-noise ratio were obtained.

## Discussion

We describe the microsurgical technique for concurrent ventral phrenic nerve dissection and cervical spinal cord access in rats. The general steps for ventral phrenic nerve dissection include midline cervical incision, removal of soft tissue and muscle, and separation of phrenic nerve from surrounding connective tissue and neural elements. The general steps of corpectomy include stepwise vessel ligation, muscle removal, tracheo-laryngeo-esophageo-pharyngeal evisceration, drilling of cervical vertebra and discs, midline cutting of dura, lateral reflection of dural leaflets, and keeping spinal cord bathed in isotonic solution. Tracheo-laryngeo-esophageo-pharyngeal evisceration is effective at preventing leaking of oral secretions into the recording mineral oil pool during spinal cord unit recordings. The critical factors differentiating favorable versus unfavorable outcome include meticulous attention while drilling bone to maintain hemostasis, as well as to avoid overheating of underlying spinal cord parenchyma. This is best achieved by performing the drilling very slowly with many small breaks between drillings and applying cold thrombin-soaked gelfoam to bleeding bone. Importantly, the dura must be cut rostrocaudally (or caudorostrally) perfectly in the midline to avoid injury to the adjacent epidural venous plexi and the dural leaflets must be gently reflected laterally. We use this approach to access phrenic motoneurons, which lie in the ventral horn of the spinal cord and are much more readily accessible for electrophysiological recordings and/or microinjections than via a dorsal approach [[3], [4], [5]]. Ventral spinal cord access has also been used to achieve cold block of the hemicord to create a functional hemisection [1,2] and can be used for a wide range of studies investigating spinal cord physiology.

## Conclusion

The ventral approach to the phrenic nerve and cervical spinal cord in rats is useful in studies investigating respiratory neurophysiology. Though requiring a more challenging surgical approach to the cervical cord, the ventrally-located phrenic motoneurons and phrenic and other respiratory-related nerves (i.e., hypoglossal, vagus, superior and recurrent laryngeal nerves) are more readily accessible for recordings. This strategy renders more facile concurrent recording of phrenic motoneurons and phrenic and other respiratory-related nerves. In contrast, the dorsal approach to accessing the cervical cord is surgically more straightforward, but a greater parenchymal distance must be traversed by recording electrodes to reach ventrally-located phrenic motoneurons and access to the phrenic nerves from this approach is slightly more challenging compared to the ventral approach.

## Methods

All procedures were approved by the Drexel University Institutional Animal Care and Use Committee, which oversees Drexel University’s AAALAC International-accredited animal program. Spontaneously-breathing, Sprague-Dawley adult male rats (350–420 g) were anesthetized with isoflurane vaporized in O_2_ (Matrix; 4–5% induction, 2.0–2.5% maintenance) via a snout mask. The electrocardiogram (EKG) was measured via three small subcutaneous electrodes using conventional amplification and filtering (Neurolog; Digitimer, Hertfordshire, UK) and monitored using an audio amplifier (model AM10; Grass Instruments) and oscilloscope. Anesthetic depth was maintained at a level such that withdrawal reflexes and changes in heart rate in response to pinches of the distal hind limbs were absent. Following tracheotomy with an atraumatic glass tube, animals were artificially ventilated with the same gas mixture (45-65 cycles/min, 2.5–3.0 ml tidal volume; Columbus Apparatus). One femoral artery and vein were cannulated for measurement of arterial pressure and infusion of drugs/fluids, respectively. During initial surgical preparation and recordings, rectal temperature was maintained at 37.0 ± 0.1 °C via a servocontrolled heating blanket coupled to a rectal thermometer (Harvard Apparatus).

**Fig. 1:**
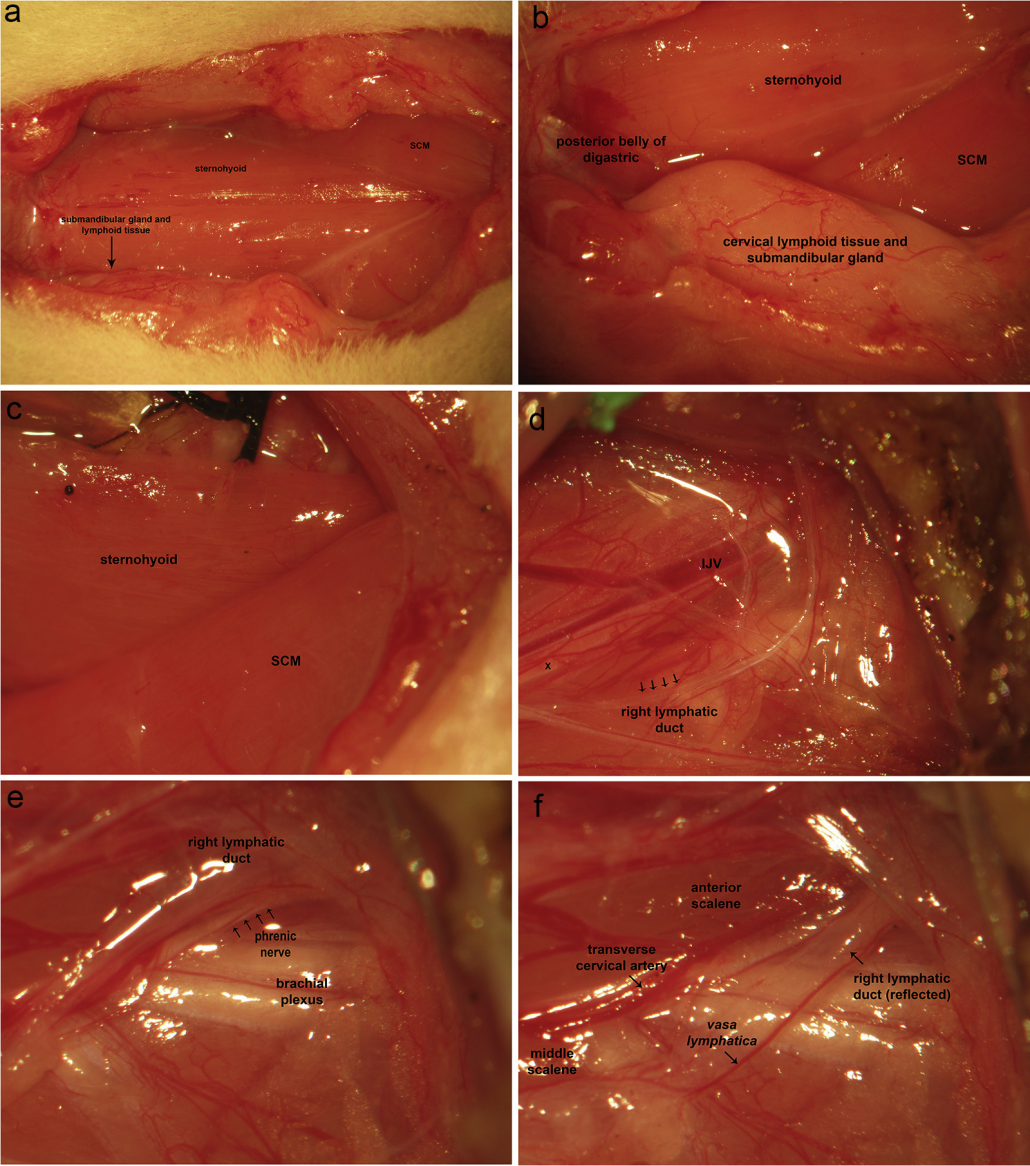

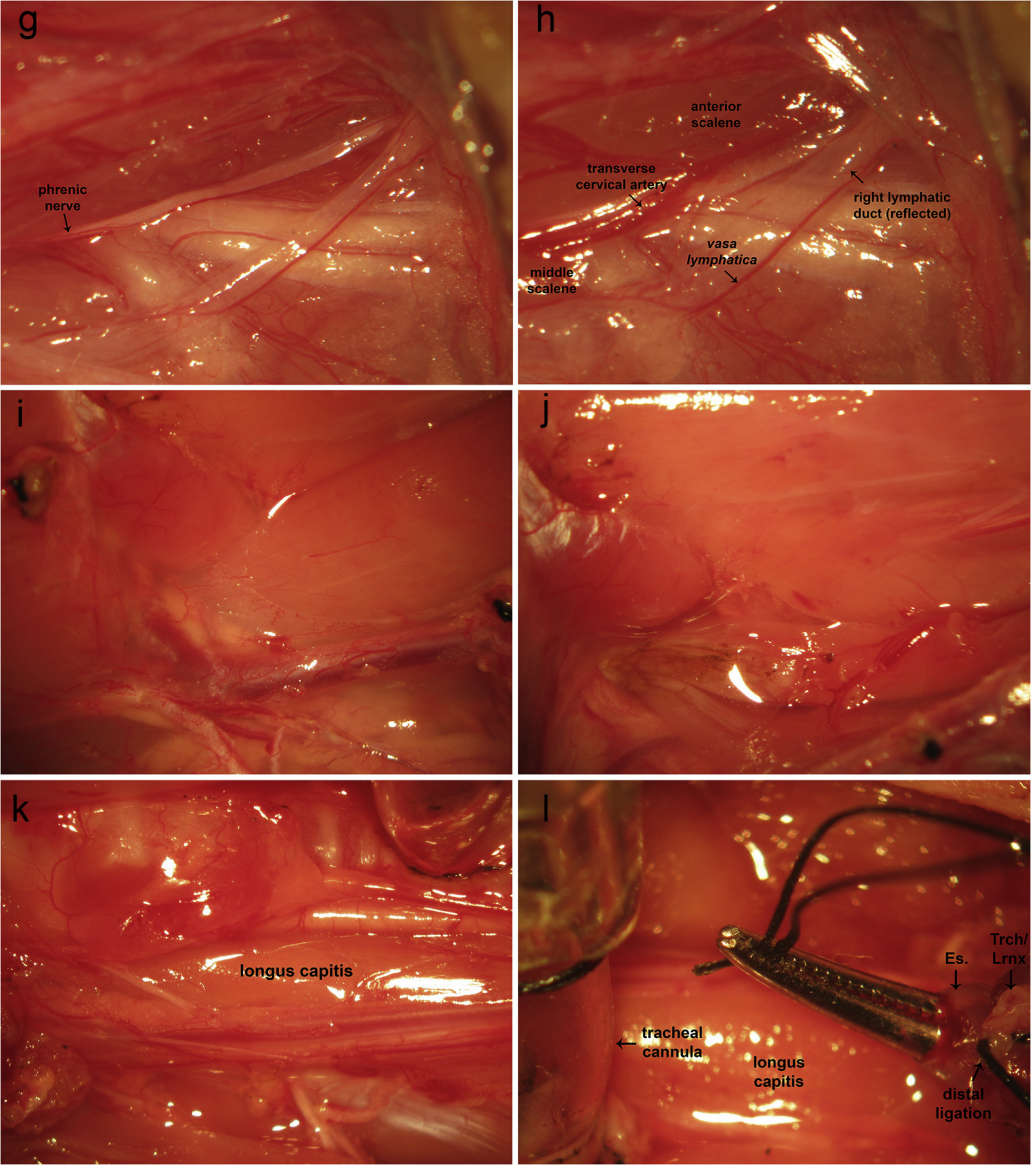

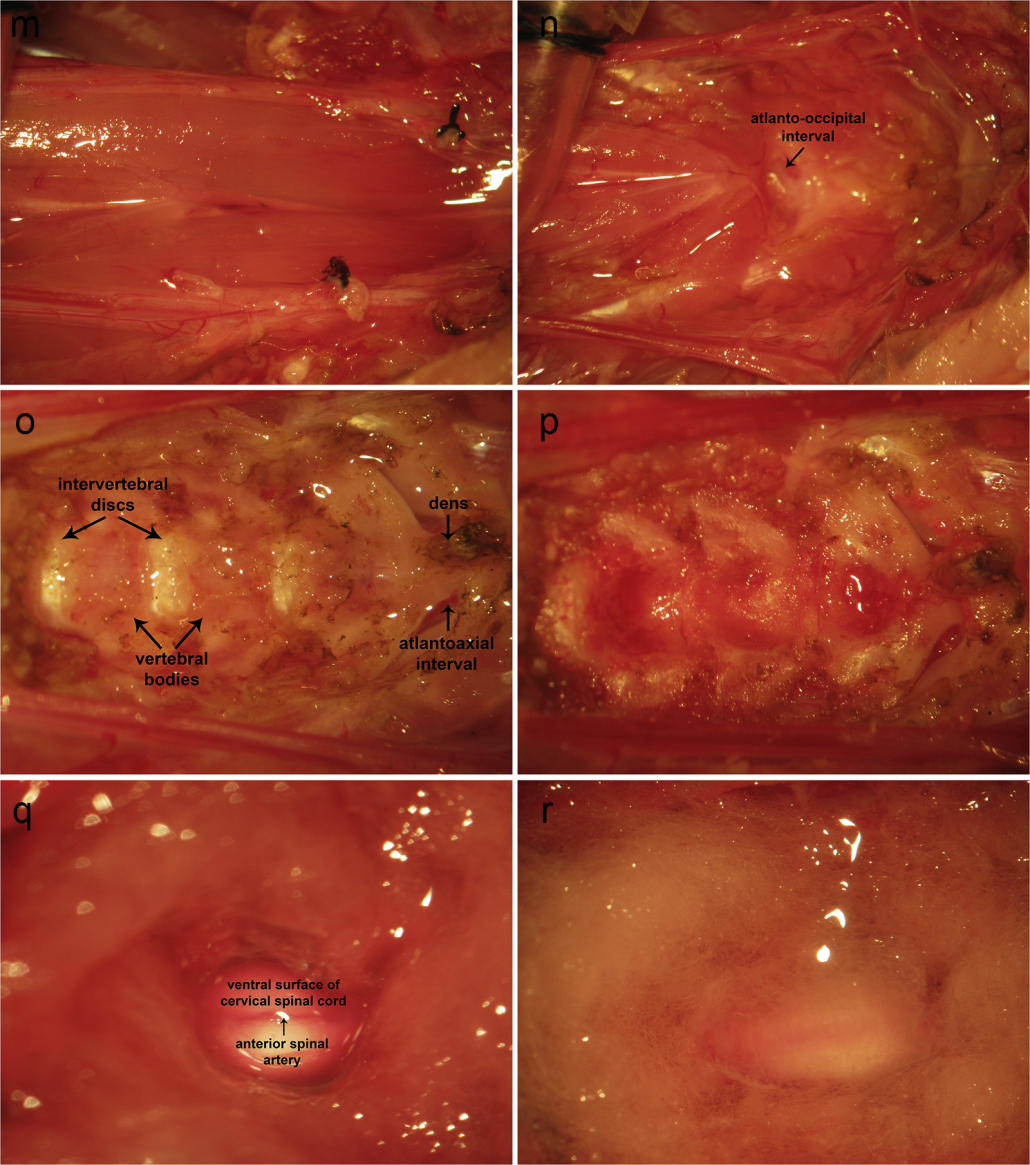
Ventral aproach to phrenic nerve access and cervical corpectomy. A: A cervical incision is made from the manubrium sterni to the level of the hyoid. Cervical lymphoid tissue is removed along with the submandibular gland. B-D: The medial origin of the sternocleidomastoid is dissected from the sternum and medial aspect of the clavicle and removed to its mastoid insertions. The sternohyoid is dissected free from its lateral origin and reflected medially and/or removed entirely. The brachial plexus region is exposed with its fascial and soft tissue investments intact. The phrenic nerve is hidden by soft tissue and the lymphatic duct with access to its proximal extent limited by the presence of the transverse cervical artery. E: Dissection of soft tissue along the lymphatic duct exposes the distal extent of the phrenic nerve. The lymphatic duct is reflected laterally, exposing the course of the phrenic nerve. F: The transverse cervical artery bridges the muscles dorsal and ventral to the phrenic nerve across the latter, concealing the proximal aspect of the phrenic nerve. The transverse cervical artery is held tightly with fine tweezers at two points so as to compress the vessel, creating stasis and promoting thrombus formation. This vessel is subsequently divided by tearing it apart with tweezers. G: This exposes the proximal extent of the phrenic nerve. Medial retraction of the scalene muscle can be employed to expose the proximal extent of the phrenic nerve to a greater extent. At this point, the phrenic nerve may be transected distally and dissected proximally to the phrenic nerve root. A 6-0 braided silk suture is used to ligate the distal end of the phrenic nerve. H-J: Previous removal of cervical lymphoid tissue, sternocleidomastoid muscle from its sternoclavicular origin to its mastoid insertion, sternohyoid, and digastric muscles, reveal the anterior jugular branch of the external jugular vein, which is double-ligated using 4-0 braided silk suture and removed. K: The trachea and esophagus are identified. L: The trachea and esophagus are securely ligated together distally and proximally leaving two long free suture ends. Forceps are inserted through the oropharyngeal cavity to the esophagus and forced through the posterior wall of the latter. The two free suture ends are grasped with a needle holder. The tracheo-laryngeo-esophageo-pharyngeal bundle is eviscerated through the oropharyngeal cavity. M: trachea-laryngeo-esophageo-pharyngeal evisceration reveals ventral musculature overlying the cervical vertebral column. The longus capiti are removed revealing the longus coli muscles. N: Removal of the longus coli reveals the ventral surface of the cervical vertebral column. O: Vertebral bodies appear sanguineous, whereas intervertebral discs are white. P: A variable-speed surgical drill is used to perform corpectomy. The richly vascularized vertebral bodies ooze blood. Use of a high-speed setting promotes hemostasis by driving bone fragments into bone marrow venous channels. Frequent pauses and irrigation with saline prevent overheating of the vertebral column and ventral surface of the spinal cord. Q: Following corpectomy, the dura is opened in the midline and carefully reflected laterally. The spinal cord is irrigated with artificial CSF and protected from surrounding bone fragments using cottonoids soaked in the same. R: A filter paper pledget may be placed over the ventral surface of the spinal cord to prevent drying of neural tissue. SCM, sternocleidomastoid. SCM, sternocleidomastoid; Es., esophagus; Trch, trachea; Lrnx, larynx; AJV, anterior jugular vein; EJV, external jugular vein.

A cervical incision is made from the manubrium sterni to the level of the hyoid. Cervical lymphoid tissue is removed along with the submandibular gland (Fig. 1A). The medial origin of the sternocleidomastoid is dissected from the sternum and medial aspect of the clavicle and removed to its mastoid insertions (Fig. 1B–D). The sternohyoid is dissected free from its lateral origin and reflected medially and/or removed entirely (Fig. 1D). The brachial plexus region is exposed with its fascial and soft tissue investments intact (Fig. 1D). The phrenic nerve is hidden by soft tissue, the lymphatic duct, and access to its proximal extent is limited by the presence of the transverse cervical artery. Dissection of soft tissue along the lymphatic duct exposes the distal extent of the phrenic nerve. The lymphatic duct is reflected laterally, exposing the course of the phrenic nerve (Fig. 1E). The transverse cervical artery bridges the muscles dorsal and ventral to the phrenic nerve across the latter, concealing the proximal aspect of the phrenic nerve (Fig. 1F). The transverse cervical artery is held tightly with fine tweezers at two points so as to compress the vessel, creating stasis and promoting thrombus formation. This vessel is subsequently divided by tearing it apart with tweezers. This exposes the proximal extent of the phrenic nerve (Fig. 1G). Medial retraction of the scalene muscle can be employed to expose the proximal extent of the phrenic nerve to a greater extent. At this point, the phrenic nerve may be transected distally and dissected proximally to the phrenic nerve root. A 6-0 braided silk suture is used to ligate the distal end of the phrenic nerve.

Previous removal of cervical lymphoid tissue, sternocleidomastoid muscle from its sternoclavicular origin to its mastoid insertion, sternohyoid, and digastric muscles, reveal the anterior branch of the external jugular vein (Fig. 1H), which is double-ligated using 4-0 braided silk suture and removed (Fig. 1I,J). The trachea and esophagus (Fig. 1K) are securely ligated together distally (Fig. 1L). The trachea and esophagus are ligated together proximally leaving two long free suture ends. Forceps are inserted through the oropharyngeal cavity to the esophagus and forced through the posterior wall of the latter (Fig. 1L). The two free suture ends are grasped with a needle holder (Fig. 1L). The tracheo-laryngeo-esophageo-pharyngeal bundle is eviscerated through the oropharyngeal cavity (Fig. 1L), revealing ventral musculature overlying the cervical vertebral column (Fig. 1M). The longus capiti are removed revealing the longus coli muscles. Removal of the longus coli reveals the ventral surface of the cervical vertebral column (Fig. 1N). Vertebral bodies appear sanguineous whereas intervertebral discs are white (Fig. 1O). A variable-speed surgical drill is used to perform corpectomy (Fig. 1P). The richly vascularized vertebral bodies ooze blood. Use of a high-speed setting promotes hemostasis by driving bone fragments into bone marrow venous channels. Frequent pauses and irrigation with saline prevent overheating of the vertebral column and ventral surface of the spinal cord. Following corpectomy, the dura is opened in the midline and carefully reflected laterally (Fig. 1Q). The spinal cord is irrigated with artificial CSF and protected from surrounding bone fragments using cottonoids soaked in the same (Fig. 1Q). A filter paper pledget may be placed over the ventral surface of the spinal cord to prevent drying of neural tissue (Fig. 1R).

## Conflicts of interest

None.

## Funding

Drexel University College of Medicine.
